# Vitamin E and Mortality in Male Smokers of the ATBC Study: Implications for Nutritional Recommendations

**DOI:** 10.3389/fnut.2020.00036

**Published:** 2020-03-31

**Authors:** Harri Hemilä

**Affiliations:** Department of Public Health, University of Helsinki, Helsinki, Finland

**Keywords:** adverse effect, alpha-tocopherol, ascorbic acid, biomarkers, evidence-based medicine, nutrition policy, nutritional requirements, reference values

## Abstract

The Dietary Reference Intakes (DRI)-monograph (USA/Canada) states that the estimated average requirement (EAR) of vitamin E for men and women of any age is 12 mg/day. The EAR value is based on *in vitro* hemolysis in young males; a surrogate endpoint without any direct validity. The EAR is then extrapolated to females and older males. The validity of the EAR level is therefore questionable. Total mortality is an outcome of direct clinical relevance. Investigating the effect of long-term dietary vitamin E intake level on mortality in a randomized trial is, however, not feasible. Nevertheless, the effect of dietary vitamin E intake can be investigated indirectly from the effects of a fixed-level vitamin E supplement administered to participants on variable levels of dietary vitamin E intake. If vitamin E intake below the EAR is harmful, then vitamin E supplement should be beneficial to those people who have dietary vitamin E intake level below the EAR. The purpose of this study was to analyze the association between dietary vitamin E intake and the effect of 25 mg/day of vitamin E supplement on total mortality in Finnish male smokers aged 50–69 years in the Alpha-Tocopherol-Beta-Carotene (ATBC) Study. The effect of vitamin E supplement was estimated by Cox regression. Among participants who had dietary vitamin C intake of 90 mg/day and above, vitamin E supplement increased mortality by 19% (*p* = 0.006) in those aged 50–62 years, but decreased mortality by 41% (*p* = 0.0003) in those aged 66–69 years. No association between vitamin E supplement effect and dietary vitamin E intake was found in these two groups, nor in participants who had dietary vitamin C intake less than 90 mg/day. There is no evidence in any of the analyzed subgroups that there is a difference in the effect of the 25 mg/day vitamin E supplement on males on dietary vitamin E intakes below vs. above the EAR of 12 mg/day. This analysis of the ATBC Study found no support for the ‘estimated average requirement' level of 12 mg/day of vitamin E for older males.

**Trial registration:** ClinicalTrials.gov, identifier: NCT00342992.

## Introduction

The Dietary Reference Intakes (DRI)-monograph of the USA and Canada states that the recommended dietary allowance (RDA) for men and women of any age is 15 mg/day. The RDA value is based on the estimated average requirement (EAR) of 12 mg/day of vitamin E [(1), p. 231–9]. This EAR value is, in turn, based on the following reasoning.

First, in 1963 Horwitt et al. had studied 4 young males and found that at plasma α-tocopherol concentrations lower than 12 μmol/L, an increase in hydrogen peroxide-induced hemolysis above 12 percent was observed *in vitro* (2). In the DRI, a plasma α-tocopherol concentration of 12 μmol/L was therefore associated with “normal” hydrogen peroxide-induced hemolysis [(1), p. 233]. Second, with young males in laboratory conditions, Horwitt had found that the plasma α-tocopherol level of 12 μmol/L was associated with the intake of 12 mg/day α-tocopherol (3), and that intake level was selected as the EAR [(1), p. 234]. Third, the authors of the DRI-monograph assumed that there were no physiological differences in vitamin E requirement between young and old adults, and concluded that “*adults ages 51 years and older appear to have the same vitamin E requirement as younger adults*” [(1), p. 238].

The above approaches are flawed. First, hydrogen peroxide-induced hemolysis in a test tube is a surrogate endpoint and the DRI-monograph does not provide any evidence that this surrogate correlates with any clinically relevant outcome [(1), p. 186–283]. The use of surrogates has been popular in medicine since measuring surrogates is much quicker and much less expensive than measuring clinically relevant outcomes. However, the wide-spread use of surrogates has been severely criticized. There are numerous examples that demonstrate that the effects on surrogate endpoints can diverge from the effects on clinically relevant outcomes (4–9). Second, several surveys in the free-living population have shown that the correlation between dietary vitamin E intake level and plasma vitamin E level is very low [(1), p. 210]. Therefore, it seems inappropriate to use a small study with males in laboratory conditions to infer a dose for males in the general population. Finally, there is much evidence that oxidative stress increases with age (10–17), and therefore, as an antioxidant, vitamin E might have different effects depending on the age of the individual. However, the authors of the DRI did not express any caution about this in the extrapolation of an EAR value derived from young males to older males and women. All three steps to establish the EAR for older people are highly speculative, and the EAR level for males aged 51 years and older is therefore extremely arbitrary. Moreover, the problems of surrogates (4–6) and the increased oxidative stress phenomenon in older people (10–12) were well known long before the DRI recommendations were published (1), and they should have been considered when deriving the EAR value. Finally, the DRI recommendations themselves admitted that there is no meaningful correlation between dietary vitamin E intake and plasma vitamin E level.

In contrast to the hydrogen-peroxide induced hemolysis in a test tube, mortality is an outcome of direct clinical relevance. Higher dietary vitamin E intake levels have been associated with lower mortality [(1), p. 212–3], but non-nutritional and nutritional confounders can bias correlations in cohort studies. Investigating the direct effect of long-term dietary vitamin E intake level on mortality in a controlled trial is not feasible. Nevertheless, the effect of dietary vitamin E intake on mortality can be investigated indirectly from the effects of a fixed-level vitamin E supplement given to subjects with variable levels of dietary vitamin E intake. If dietary vitamin E intakes below the stated EAR are harmful, then vitamin E supplement should be beneficial to people with vitamin E intakes below the EAR.

The purpose of this study was to analyze the association between dietary vitamin E intake and the effect of vitamin E supplementation on mortality in the Alpha-Tocopherol-Beta-Carotene (ATBC) Study. The ATBC Study was a large randomized trial, which examined the effects of 50 mg/day of *all rac*-α-tocopheryl acetate and 20 mg/day β-carotene on the risk of lung cancer in male smokers aged 50–69 years (18, 19). The *all rac*-α-tocopheryl acetate has only 50% of the vitamin E activity as redefined in the DRI in 2000 [(1), p. 191], which indicates that the actual biologically active supplement dose was 25 mg/day of the redefined vitamin E.

A previous analysis of the ATBC Study data showed that the effect of vitamin E supplement on mortality was significantly modified by the combination of age and dietary vitamin C intake (20). The effect of vitamin C was not explained by other substances in fruit and vegetables (20). Old age is associated with increased oxidative stress (10–17) and there is strong evidence of an interaction between vitamins C and E both *in vitro* and *in vivo* (21–30), which makes these two variables plausible modifiers of the vitamin E supplement effects (20). If the EAR level of 12 mg/day is a biologically valid estimate, then the effects of vitamin E supplementation should be different for ATBC participants with vitamin E intakes below and above the EAR level in the previously identified groups defined by age and dietary vitamin C intake.

## Methods

### Participants

The design and methods of the ATBC Study examining the effects of vitamin E [*all rac*-α-tocopheryl acetate, AT, 50 mg/day, which corresponds to 25 mg/day of redefined vitamin E [(1), p. 191] and β-carotene (BC, 20 mg/day) on the incidence of lung cancer and other cancers have been described earlier (18, 19). The ATBC Study is registered at ClinicalTrials.gov under the identifier NCT00342992.

In brief, male participants aged 50–69 years had to smoke ≥5 cigarettes per day at entry to be eligible, and those enrolled in the trial (*N* = 29,133) were randomized to one of four intervention arms and administered placebo, AT, BC or AT + BC, using a 2 × 2 factorial design. Compliance with supplementation was high: 90% of the participants took more than 90% of their prescribed capsules during their active participation in the trial; there were no differences in capsule consumption among the intervention groups. Compared with the baseline levels, supplementation increased the serum level of α-tocopherol by 50%. The intervention continued until 30 April 1993. The trial was approved by the institutional review boards and all participants gave written informed consent. This study was restricted to 27,111 participants for whom dietary data were available.

### Baseline Characteristics

Before randomization at baseline, the men completed questionnaires on their general background characteristics. At the first baseline visit, participants were given a separate, detailed dietary history questionnaire for completion at home and the questionnaire was returned and reviewed (31). The questionnaire included a 63-page picture booklet with 122 photographs, and asked about portion sizes for 276 common foods and mixed dishes and the usual frequency of their consumption over the previous 12 months. The validity of the dietary history questionnaire was assessed by comparing it with the food consumption records of 190 participants for 12 separate 2-day periods distributed evenly over 6 months. Between 75% and 80% of participants categorized by food consumption records obtained from the dietary history questionnaire were in the same vitamin E intake quintile or in the within-one-quintile category, and 74–76% were in the same vitamin C intake quintile or in the within-one-quintile category (31). In the reproducibility study, participants filled in the food use questionnaire three times, at three-month intervals. The intraclass correlation was 0.70 for dietary vitamin E intake and 0.69 for dietary vitamin C intake as determined by the reproducibility analysis (31).

Dietary data were not available for 2,022 of the 29,133 participants. In the ATBC Study, dietary vitamin E intake was estimated as α-tocopherol equivalents. The DRI monograph instructs using a multiplier 0.80 for the transformation of α-tocopherol equivalents to the redefined vitamin E dose [(1), p. 244–5], however, the multiplier of 0.85 was used in this study as it was based on Finnish data (32). Using the multiplier 0.80 has no effect on the trend tests, and has only minor effects in the groups by dietary vitamin E intake (not shown).

### The Vitamin E Variables

In this analysis, the term “vitamin E intake” refers to the dietary vitamin E intake at the baseline of the ATBC Study (31). The term “vitamin E supplementation” refers to the administration of 25 mg/day of the redefined vitamin E as *all rac*-α-tocopheryl acetate (50 mg/day) [(1), p. 191]. Thus, the total daily intake of vitamin E among the vitamin E participants was the sum of the dietary vitamin E intake plus the supplement dose of 25 mg/day.

### Outcome and Follow-up Time

Deaths were identified in the National Death Registry as previously described (18, 19). Follow-up time for each participant began from the day of randomization, and continued until death or the end of the trial (30 April, 1993). The median follow-up time of the participants in the present analysis was 6.1 years, and there was a total of 158,373 person-years of observation.

### Statistical Models

The effect of vitamin E supplementation on mortality was estimated through Cox regression models. The risk ratio (RR) and the 95% confidence interval (CI) of the RR were calculated using PROC PHREG in SAS (release 9.4, SAS Institute, Inc., Cary, NC, USA). Participants administered vitamin E (AT and AT+BC) were compared with those not administered vitamin E (the no-vitamin E participants; placebo and BC).

The potential modification of vitamin E supplement effect by dietary vitamin E intake, and by β-carotene supplementation was examined as follows. To test the statistical significance of interaction between vitamin E supplement and a potential modifying factor, the supplement and modifying factor were first added to the Cox regression model. Then the interaction term was added to the model. The statistical significance of the interaction was thereafter calculated by using the likelihood ratio test, see Supplementary Material. As to supplementation, the analyses were carried out following the intention-to-treat principle. Deaths were identified in the National Death Registry which registers all deaths that occur in Finland, thus the loss-to-follow-up is insignificant (18, 19).

## Results

The Alpha-Tocopherol-Beta-Carotene (ATBC) Study recruited 29,133 male smokers aged 50–69 years. The characteristics of the participants have been described previously (18, 19, 33). Among all participants, vitamin E had no average effect on mortality (19). Dietary data for vitamins C and E were available for 27,111 participants to whom this analysis is restricted. An earlier analysis showed that dietary vitamin C intake and age modified the effect of vitamin E supplementation (20), and the following analysis is an extension of those previous findings. Since vitamin E supplement effect was different for males who had high and low dietary vitamin C intake, the participants are divided here into two subgroups by the vitamin C intake of 90 mg/day, which is close to the median.

Vitamin E supplement had no effect on mortality in participants who had vitamin C intake <90 mg/day. There was no variation in vitamin E supplement effect by dietary vitamin E effect (Table 1). There is no evidence that vitamin E supplement was beneficial for the 11,223 participants who had dietary vitamin E intake less than the stated EAR of 12 mg/day (1), with RR = 1.00 (95% CI 0.91-1.11; based on 735/736 deaths in the vitamin E and no-vitamin E groups). Stratification by age is not shown in Table 1 since the lack of vitamin E supplement effect is uniform over the three age groups.

**Table 1:** ATBC Study participants with dietary vitamin C intake <90 mg/day by dietary vitamin E intake.

	**Dietary vitamin E intake**			**Test of trend^a^*P***
Range (mg/day)	1.4–8	9–11	12–58	
Median intake (mg/day)	6.5	10.1	15.3	
Vitamin E supplement effect^b^ RR (95% CI)	1.03 (0.92–1.16)	0.92 (0.73–1.15)	1.01 (0.81–1.26)	0.5
Deaths (vitE/no-vitE)	588/579	147/157	160/156	
*N*	8740	2483	2344	

a *Test of interaction between vitamin E supplement effect and dietary vitamin E intake as a continuous variable. The linear trend was tested and the p > 0.05 value indicates that there is no evidence that the vitamin E supplement effect varies by dietary vitamin E intake level*.

b *The Cox regression model comparing participants who received vitamin E with those who did not. For the 11,223 participants who had dietary vitamin E intake below 12 mg/day (median 7.1 mg/day), which is the EAR level for ages 51 years and older [(1), p 238], the RR = 1.00 (95% CI 0.91–1.11)*.

*RR, risk ratio; CI, confidence interval*.

In those who had baseline vitamin C intake 90 mg/day and above, the effect of vitamin E supplement was modified by age (Table 2). Within the three age groups there was no evidence that vitamin E supplement effect was modified by dietary vitamin E intake. The confidence intervals extensively overlap and the tests of interaction between vitamin E supplement and continuous dietary vitamin E intake found no significant associations (Table 2).

**Table 2:** ATBC Study participants with dietary vitamin C intake ≥90 mg/day by dietary vitamin E intake.

	**Dietary vitamin E intake**			**Test of trend^a^*p***
Range (mg/day)	3.1–8	9–11	12–53	
Median intake (mg/day)	7.5	10.3	15.2	
**Age 50–62 years**				
Vitamin E supplement effect^b^				
RR (95% CI)	1.15 (0.93–1.41)	1.23 (0.98–1.54)	1.20 (0.97–1.47)	0.3
Deaths (vitE/no-vitE)	188/167	168/134	196/168	
*N*	3591	3558	4299	
**Age 63–65 years**				
Vitamin E supplement effect^b^				
RR (95% CI)	1.17 (0.77–1.75)	0.78 (0.46–1.31)	0.69 (0.42–1.13)	0.09
Deaths (vitE/no-vitE)	52/42	27/29	27/39	
*N*	498	350	376	
**Age 66–69 years**				
Vitamin E supplement effect^b^				
RR (95% CI)	0.59 (0.37–0.92)	0.78 (0.46–1.31)	0.40 (0.22–0.74)	0.7
Deaths (vitE/no-vitE)	30/52	27/31	14/41	
*N*	360	263	249	

a *Test of interaction between vitamin E supplement effect and dietary vitamin E intake as a continuous variable. The linear trend was tested and p > 0.05 value indicates that there is no evidence that the vitamin E supplement effect varies by dietary vitamin E intake level*.

b *The Cox regression model compares participants who received vitamin E supplementation with those who did not*.

*RR, risk ratio; CI, confidence interval*.

**Figure 1:**
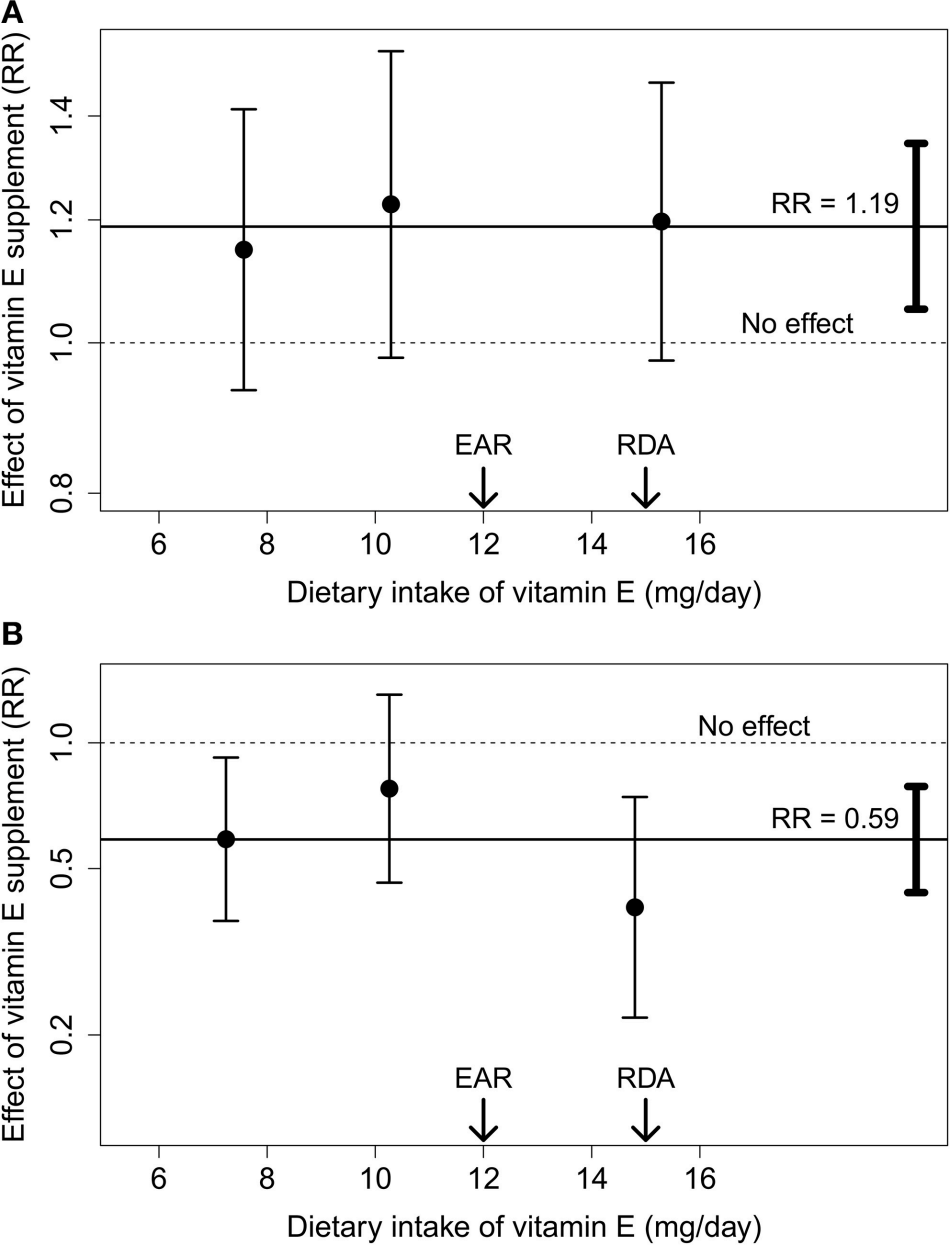
The effect of vitamin E supplement by dietary vitamin E intake. **(A)** Participants aged 50–62 years at baseline. **(B)** Participants aged 66–69 years at baseline. The three vitamin E intake groups of Table 2 are plotted at the median vitamin E intake of the respective group. The points indicate the estimates and the vertical lines indicate their 95% CIs. The solid horizontal lines indicate the average effect of vitamin E supplement in the younger **(A)** and older **(B)** participants, and the vertical bars on the right-hand side indicate the 95% CI for the average effect. The dashed horizontal lines indicate the no effect level. The arrows indicate the levels of the EAR and the RDA for vitamin E (1).

Vitamin E supplement was significantly harmful for the younger ATBC participants aged 50–62 years who had vitamin C intakes of 90 mg/day and above (Table 2 and Figure 1A). On average, the 25 mg/day vitamin E supplement increased mortality by RR = 1.19 (95% CI 1.05–1.35; *p* = 0.006). There is no evidence that vitamin E supplement was beneficial for the 7,149 participants who had dietary vitamin E intake less than the EAR of 12 mg/day (1), with RR = 1.18 (95% CI 1.01–1.38; *p* = 0.034; based on 356/301 deaths in the vitamin E and no-vitamin E groups). The median vitamin E intake was 9.0 mg/day in the 7,149 participants who had intake below the EAR.

In the older ATBC participants aged 66–69 years who had vitamin C intake 90 mg/day and above, vitamin E supplement was significantly beneficial (Table 2 and Figure 1B). On average, vitamin E supplement decreased mortality by RR = 0.59 (95% CI 0.44–0.79; *p* = 0.0003). A statistically significant benefit of vitamin E supplementation was seen in those who had dietary vitamin E intake of <9 mg/day and also in those who had dietary vitamin E intake 12 mg/day and above. The confidence interval for the middle group (10 mg/day) extensively overlaps with the groups on the sides. The significant benefit of vitamin E supplementation in the right-hand-side group indicates that the median intake of 15 mg/day was sub-optimal, although 15 mg/day is the RDA level and has been argued to cover the requirement of 97.5% of people [(1), p. 23,238].

Since half of the ATBC Study participants were given β-carotene and half were not, the possible interaction between β-carotene and vitamin E administration was examined in the three age groups who had vitamin C intake of 90 mg/day and over (Table 3). There was no evidence found that the vitamin E supplement effect was modified by the β-carotene supplement. In the younger participants aged 50–62 years, the point estimates for harm of vitamin E supplement were very similar in both groups. In the older participants aged 66–69 years group, the estimates for benefit were also very similar. Thus, the estimates of effect in Table 2 are not biased by β-carotene administration.

**Table 3:** ATBC Study participants with dietary vitamin C intake ≥90 mg/day by β-carotene administration.

	**No-β-caroteneparticipants**	**β-Caroteneparticipants**	**Test of interaction^a^*p***
**Age 50–62 years**			
Vitamin E supplement effect^b^			
RR (95% CI)			
	1.20 (1.00–1.44)	1.17 (0.99–1.39)	0.9
Deaths (vitE/no-vitE)	259/222	293/247	
*N*	5710	5738	
**Age 63–65 years**			
Vitamin E supplement effect^b^			
RR (95% CI)	0.76 (0.52–1.11)	1.04 (0.71–1.53)	0.3
Deaths (vitE/no-vitE)	50/60	56/50	
*N*	609	615	
**Age 66–69 years**			
Vitamin E supplement effect^b^			
RR (95% CI)	0.57 (0.38–0.86)	0.60 (0.39–0.91)	0.9
Deaths (vitE/no-vitE)	37/59	34/65	
*N*	429	443	

a *Test of interaction between vitamin E supplementation and β-carotene supplementation. The p > 0.05 value indicates that there is no evidence that the vitamin E supplement effect is influenced by β-carotene supplementation in these groups*.

b *The Cox regression model compared participants who had received vitamin E with those who did not separately in the no-β-carotene participants and the β-carotene participants*.

*RR, risk ratio; CI, confidence interval*.

**Figure 2:**
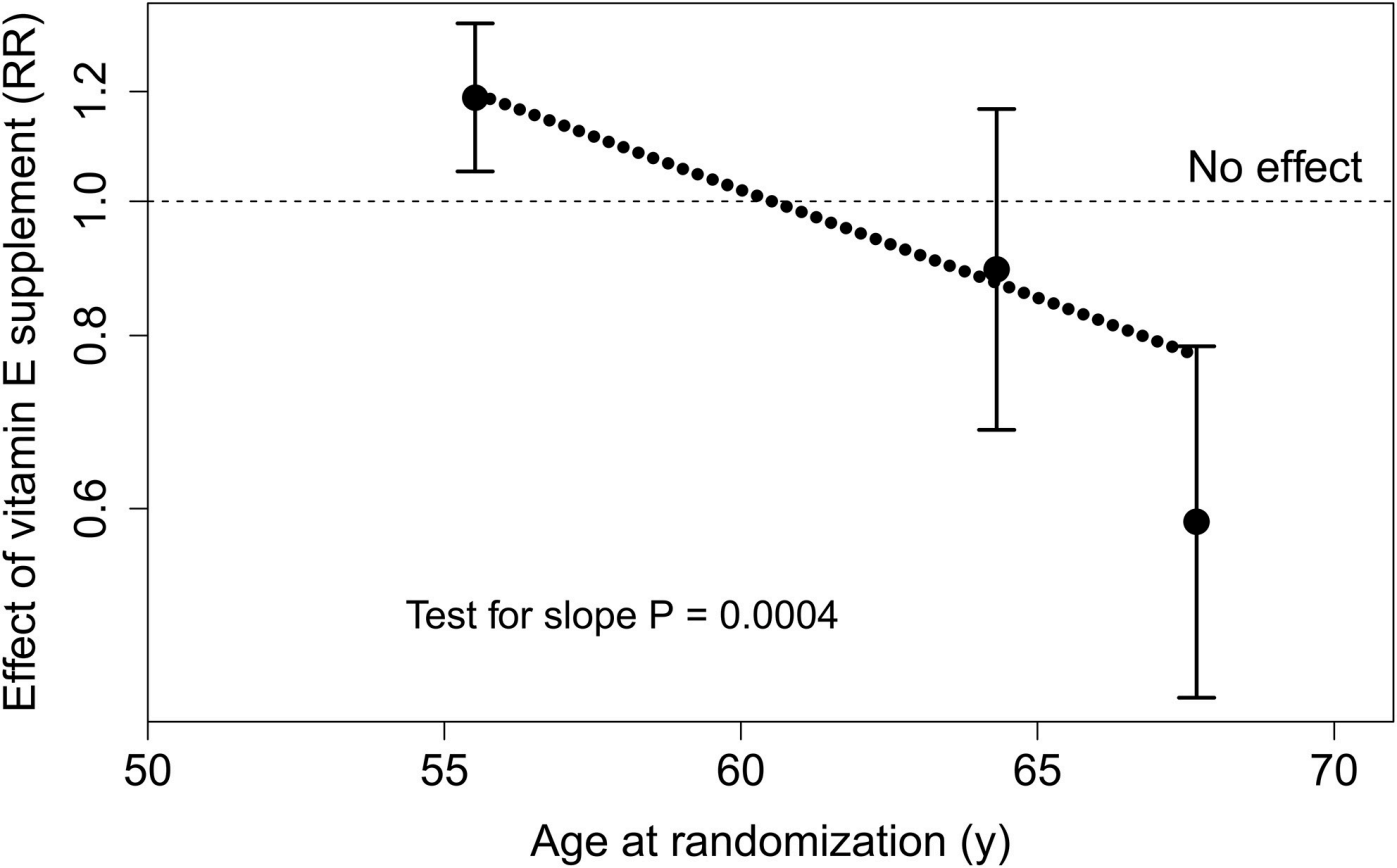
Relationship between baseline age and the effect of vitamin E supplement in males who had dietary vitamin C intake ≥90 mg/day. The three age groups of Table 2 are plotted at the median age of each group. The points indicate the estimates and the vertical lines indicate their 95% CI. The uniformity of the vitamin E supplement effect over age was tested by adding a dummy variable for vitamin E effect for the 63–65 year and the ≥66 year age groups, allowing each of the three age groups of Table 2 their own vitamin E effect. The regression model was improved by $\chi_{\left(2\text{df}\right)}^{2}$ = 21.5, *p* = 0.00002, compared with the model with a uniform vitamin E supplement effect over all ages. The dotted diagonal line shows the Cox regression model with age as a continuous variable. Allowing the slope, the regression model was improved by $\chi_{\left(1\text{df}\right)}^{2}$ = 12.8, *p* = 0.0004, compared with the model with a uniform vitamin E supplement effect over all ages.

Among the participants who had vitamin C intake 90 mg/day and above, the relationship between age and vitamin E supplement effect is shown in Figure 2. Allowing each of the three age groups of Table 2 their own vitamin E supplement effect improves the Cox regression model significantly. However, age was also analyzed as a continuous variable since interaction between vitamin E supplement effect with continuous age refutes the possibility that explorative selection of the cut-points might have caused a spurious interaction with age. The dotted diagonal slope in Figure 2 tests whether the effect of vitamin E supplement is constant over age, and a uniform effect over age as a continuous variable is rejected. The regression line should not be extrapolated beyond the median vitamin E intakes of the youngest and oldest participants, but the slope indicates that there are substantial changes in vitamin E effects in the age region of ~60–65 years.

## Discussion

### Main Findings

No evidence was found to indicate that the effect of vitamin E supplementation might differ for those who had dietary vitamin E intake below vs. above the EAR level for vitamin E. This lack of difference in the vitamin E supplementation effect below and above the EAR level was seen separately in four subgroups of the ATBC cohort. In two of the subgroups, vitamin E supplementation had a significant effect on mortality, but the effect was closely similar on both sides of the EAR limit. In the older participants who had vitamin C intakes over 90 mg/day and dietary vitamin E intake over 12 mg/day, the vitamin E supplement was significantly beneficial even though the median dietary vitamin E intake in that subgroup was 15 mg/day, which is the RDA (2000) level for vitamin E (1). Therefore, the dietary vitamin E intake of that subgroup should cover the requirement of 97.5% of people [(1), p. 23,238]. The significant variation in vitamin E supplement effect by age and dietary vitamin C intake in the males of the ATBC cohort is inconsistent with a uniform EAR level for all adults.

### Three Alternative Paradigms on Vitamins and Health

For the interpretation of the current findings, it is useful to consider three different paradigms that have been proposed for the relationship between vitamin intake levels and health.

Many vitamins were found as the explanation for a severe disease, such as the deficiency of vitamin C was identified as the explanation for scurvy. This background led to the ‘nutrient need' -paradigm (34–37). Preventing scurvy was the premise of the US RDA nutritional recommendations for vitamin C from the 1940s until the 10th edition in 1989 (38, 39). Approximately 10 mg/day of vitamin C prevents overt symptoms of scurvy, and this level was termed the “need” or “requirement.” Nevertheless, the concept of ‘nutrient need' gives the false impression that an exact dose of a vitamin is required daily, so that larger amounts could not have any active physiological effects, but would simply provide passive reserves. However, the physiological purpose of vitamin C is not to remain “in reserve against scurvy” but to participate in chemical reactions in the body, and the rates of these reactions depend on concentrations, which further depend on the levels of intake (34–37, 40–44). Thus, an exact level of “nutrient need” or “requirement” that sharply distinguishes a frank deficiency from “normal health” is not a sound concept from the biochemical point of view. The RDA for vitamin C (1989) was set at 60 mg/day to “*provide an adequate margin of safety*” against scurvy, arguing that “*this level of intake will prevent signs of scurvy for at least 4 weeks*” in the fictional condition that a person completely stops taking any vitamin C [(38), p. 118].

The “optimal intake” -paradigm also has its origins in the early part of the previous century. Charles Glen King (1936) commented that “*The fact that there is a wide zone of vitamin deficiency between scurvy and optimum health is of more interest in relation to human health than the problem of clinical scurvy*” [(45), p. 254]. From his own research on guinea pigs he also concluded that “*it is evident from the data presented that the level of vitamin C intake for optimum in vivo detoxification of diphtheria toxin is considerably greater than that necessary to protect from scurvy or to show a favorable growth rate*” [(46), p. 8]. The optimal intake approach was further elaborated by Linus Pauling in the late 1960s (47), and later by other authors (34–36, 41–44, 48–50). The optimal intake -paradigm is consistent with gradual changes in the rates of biochemical reactions with changes in concentrations. Evidently, the optimal intake is not a sharp peak, but a wide plateau. However, in some contexts the plateau might start from dose levels that are much higher than those needed to prevent overt deficiencies. In the RDA (1989), the lack of concern about optimal intakes was explicitly recognized: “*RDAs are not necessarily optimal levels of intake”* [(38), p. 8]. It seems highly likely that the long-term optimal levels are different from the doses that make survival possible in the short term, i.e., doses that prevent frank deficiency diseases (14, 16, 49, 50). The term “optimal requirement” has been used (42, 43), but that is a misnomer, since the term “requirement” has a long and strong history of indicating the minimal dose that prevents overt deficiency. In contrast, “optimal intake” or “optimal dosage” seem more accurate terms that do not have similar historical load.

The third view on the vitamin intake levels and health is the “individuality in nutrition” -paradigm proposed by Roger J Williams (51–53). The first two paradigms detailed above are not inconsistent with individuality. The view of the DRI is that “requirement” has a normal distribution in the population, with the EAR as its mean with the standard deviation of 10% of the EAR [(1), p. 23–4,237–9]. However, Williams considered that people might differ much more greatly so that their individual differences in requirement are not simply a marginal variation around the mean of a normal distribution. Individuality in nutrition can have genetic origins (54). One mechanistic explanation for substantial differences between people in their individual optimal levels of vitamin intake can be due to dissimilarities in the binding constants of enzymes (47, 48). In addition, life style factors and characteristics such as age may influence the optimal intake levels.

According to Thomas Kuhn, a paradigm defines what kinds of questions may be asked and what kinds of answers are plausible (55). One illustration of the influence of a paradigm in guiding argumentation in nutrition is the topic of vitamin C and the common cold. In 1971, Pauling published a meta-analysis in which he showed that there was highly significant evidence from four placebo-controlled trials that vitamin C is beneficial against the common cold with *p* = 0.00002 (56), a conclusion that has since been corroborated in a large series of further randomized trials (57, 58). However, the RDA (1989) recommendations (38) did not base the conclusions on vitamin C and the common cold on the Pauling meta-analysis, but instead on the meta-analysis by Thomas Chalmers which concluded that there was no evidence that vitamin C had effects on colds (59). Nevertheless, the Chalmers meta-analysis is flawed as it has errors in data extraction, in the calculations, and it is inconsistent in its inclusion criteria for trials (37, 60, 61). In contrast, errors have not been pointed out in Pauling's analysis (56). Had the authors of the RDA monograph taken any look at the original publications they would have seen at least some of the problems in the Chalmers meta-analysis. So, why did they ignore the Pauling meta-analysis? This may be explained by confirmation bias which indicates the tendency to search for and interpret information in a way that confirms one's prior beliefs (62). This serves as an example how the “nutrient need” paradigm, e.g., the belief that vitamin C has effects only on scurvy, can blind authorities from objectively looking at evidence that had shown already by 1971 that vitamin C can influence conditions other than scurvy. Kunkel has also discussed how belief systems have affected the development of RDAs and nutrition guidelines in the past and he expected them to do so also in the future (63).

### Are Current Vitamin E Recommendations Appropriate?

The above three paradigms provide a useful conceptual background for considering the current findings and the vitamin E recommendations. Overt vitamin E deficiency symptoms have never been described in normal individuals consuming diets low in vitamin E because the vitamin is found in such a great variety of foods and it is subsequently stored in the body for extensive periods. Therefore, the “nutrition need” paradigm is not applicable to vitamin E because there is no vitamin E deficiency in the same sense as scurvy is the deficiency of vitamin C. In the RDA (1989), the approach for vitamin E was pragmatic. Since there was no evidence of vitamin E deficiency in the general population, the quantities that people usually obtain from their diet cannot be too low. This reasoning was the basis for the RDA (1989): “*the allowance, therefore, is based primarily on customary intakes from U.S. food sources… an arbitrary but practical allowance for male adults of 10 mg of [*α*-tocopherol equivalents] per day*” [(38), p. 103].

In contrast, the EAR for vitamin E in the DRI (2000) recommendations is based on hydrogen peroxide-induced hemolysis *in vitro*, which is a surrogate endpoint without proven validity to any clinically relevant outcome [(1), p. 232–9]. Furthermore, the authors of the DRI recommendations believed that the distribution of the “requirement” follows the normal distribution and that the standard deviation (SD) is 10% of the EAR [(1), p. 23–4,237–9]. In a normal distribution, the mean plus 2 SD covers 97.5% of the distribution, and such a level was labeled as the revised RDA (2000) level: “*The Recommended Dietary Allowance (RDA) is the average daily dietary intake level that is sufficient to meet the nutrient requirement of nearly all (97 to 98 percent) apparently healthy individuals*” [(1), p. 23]. For vitamin E, this led the authors of the DRI monograph to calculate that vitamin E intakes at 15 mg/day (i.e., 12 mg/day + 20%) would cover the “requirements” of essentially all of the general population. However, there was no scientific basis for the assumption that the distribution of ‘requirement' for vitamin E might have a SD of 10% (1). Beaton heavily criticized the assumption of the 10% SD in the calculation of the RDA (2000): “*the ‘scientific basis' and hence validity of RDAs calculated with uncertain or assumed variation and unknown distribution characteristics must be questioned*” (64, 65).

Table 1 of the current study challenges the validity of the 12 mg/day as a biologically relevant limit. There was no benefit from the 25 mg/day vitamin E supplement to the 11,223 low-vitamin C-participants who had dietary vitamin E intakes less than the EAR of 12 mg/day. Table 2 of the current study also contradicts with the 12 mg/day as a valid limit. There is no evidence in any of the three age groups that there is a difference in the effect of the 25 mg/day vitamin E supplement on males on dietary vitamin E intakes below vs. above the EAR of 12 mg/day. Thus, these current findings challenge the status of the 12 mg/day as being a biologically valid “average requirement” level for males aged 51 years and older.

Although the DRI claims that 15 mg/day would cover the “requirements” of 97.5% of 51 year-olds and older [(1), p. 238], the administration of an additional 25 mg/day of vitamin E to the 249 older high-vitamin C participants who already had a median dietary vitamin E intake of 15 mg/day (i.e., the new RDA), had a significantly decreased mortality (Table 2). The comparison within this group was between the dietary vitamin E intake level of 15 mg/day in the no-vitamin E participants and the total intake of 40 mg/day (= 15 + 25 mg/day) in the vitamin E participants. Thus, the 12 mg/day as the “estimated average requirement” of vitamin E for males aged 51 years and older is based on flawed approach and it is contradicted by empirical data from the ATBC Study, which found no evidence that such a level has biological relevance.

The current findings resonate particularly well with the “individuality in nutrition” -paradigm. The distribution of vitamin E effects in Tables 1, 2 are inconsistent with one single level of “average requirement” that applies to the general population so that there is a normal distribution -type variation around a uniform mean [(1), p. 23–4]. In contrast, male smokers of different ages with different vitamin C intakes seem to have quite a different relationship between the vitamin E dosage and health effects. In the ATBC Study, very strong evidence of heterogeneity was also shown for the effects of vitamin E supplement on the incidence of pneumonia (33, 66–69) and the common cold (70). These findings further contradict a universal “average requirement” level for vitamin E.

Although Table 2 gives information about vitamin E dose dependency in the three age groups, the confidence intervals are wide and the data points are too sparse for drawing detailed conclusions about the optimal intake range. Evidently, the optimal range for the younger males of 50–62 years with high vitamin C intake is less than 34 mg/day since the administration of 25 mg/day of vitamin E above the median daily intake of 9 mg/day led to harm (Table 2). The optimal intake in the older males of 66–69 years with high vitamin C intake appears to be over 15 mg/day, but there is no basis to conclude whether the optimum for these individuals might be below or above 40 mg/day (= 15 + 25 mg/day) (Table 2).

Figure 2 indicates that there are changes in the effects of vitamin E in the age region of 60–65 years. Great changes in the effects of vitamin E and β-carotene supplementation on the incidence of the common cold were also observed in the age range of 60–65 years (70, 71). In addition, the effects of vitamin E and β-carotene on pneumonia incidence (69, 72) and the effect of vitamin E on mortality (73) were most evident in the oldest age range at follow-up.

### Adverse Effects of Increased Intakes of Vitamin E

The DRI recommendations concluded that the tolerable upper intake level (UL) for vitamin E is 1.0 g/day [(1), p. 255–8]. This estimate was based on studies on rats, but it is possible that harms in rats have very different dose-responses than harms in humans. For example, various life style variables such as smoking, alcohol intake, and physical activity, and also characteristics of humans such as age and blood pressure, and the duration of supplementation of the fat-soluble vitamin might modify the potential harms of increased intakes of vitamin E. The effects of such variables cannot be reasonably tested in rats.

The increased mortality in the younger 50–62 year-old participants (Table 2) of the present study started only after a lag period of about 3 years, possibly because fat-soluble vitamin E accumulates slowly (20). In addition to the harm shown for the younger participants in Table 2, the 0.05 g/day α-tocopherol supplement in the ATBC study increased the risk of fatal subarachnoid hemorrhage by 181% (*p* = 0.005) (74). That harm was most evident in participants with hypertension in whom fatal subarachnoid hemorrhages increased by 438% (*p* = 0.03) (75). Vitamin E supplementation was also associated with a 14% higher risk of pneumonia in ATBC participants who initiated smoking at an early age (33), but that harm was further modified by current smoking, lack of leisure-time exercise, body weight and dietary vitamin C intake, all of which defined groups in which significant harm occurred (66–68). Finally, vitamin E increased the risk of tuberculosis by 120% in ATBC participants who smoked heavily and had a high dietary intake of vitamin C (76, 77). These findings illustrate that studies on rats can be highly misleading on the potential adverse effects of long-term vitamin E administration in particular population groups.

Individual-level analysis is most informative in the investigation of particular conditions and harms associated with increased vitamin E intakes. However, significant study-level harm has also been reported in other randomized trials. A study on 652 Dutch elderly people found that 0.2 g/day of vitamin E increased the occurrence of fever with respiratory infections (*p* = 0.01) and the total illness duration (*p* = 0.02) (78). The HOPE trial found that 0.3 g/day of vitamin E increased the risk of hospitalization for heart failure by 40% (*p* = 0.002) in 7,030 participants with the longest treatment and follow-up (79). The Physicians' Health Study II found that 0.4 g/day of vitamin E increased hemorrhagic stroke by 17% (*p* = 0.04) in 14,641 male physicians (80). The SELECT trial found that 0.4 g/day of vitamin E increased the incidence of prostate cancer by 17% (*p* = 0.01) in 17,433 males (81). All these doses are much lower than the rat-based UL of 1.0 g/day for humans [(1), p. 255–8]. Furthermore, it is noteworthy that the increase in prostate cancer incidence started after about a 3-year lag period (81), which is consistent with the few years lag that was observed before the increase in mortality in the young ATBC Study subgroup (20).

### Critique of the DRI Approach by Max Horwitt

The two trials on which the EAR level for vitamin E were based, were carried out by Max Horwitt (2, 3). Interestingly, Horwitt seriously criticized the approach in the calculation of the EAR (82). As early as 1963 Horwitt had stated that he had requested that no specific requirement for vitamin E be adopted on the basis of his studies. He emphasized that the experimental diets in the studies had contained large amounts of oxidized unsaturated fats not found in habitual diets (82). He also pointed out that millions of people have lived long lives while consuming much less that 10 mg/day of vitamin E. Thus, according to his opinion, increasing the assumed ‘requirement' to 15 mg/day “*benefits only the commercial interests involved in the sale of vitamin E*.” Horwitt was also concerned with setting the tolerable upper intake level to 1 g/day. He noted that millions of persons swallowed aspirin each day, and there was already at that time evidence that vitamin E could enhance the anticoagulant effects of aspirin (83). After Horwitt's critique there has emerged much stronger evidence that vitamin E doses substantially lower than 1 g/day can cause significant harm in some population groups (20, 66–68, 74–81).

### Limitations of the Study

The ATBC participants were all male smokers and they were born before WWII. Thus, their childhood and youth were very different compared to those of later generations. In that respect, it is not evident how far various findings of the ATBC cohort can be extrapolated to the general current Western population. However, having such a background does not compromise the use of the ATBC cohort for the examination of the validity of the EAR level. The EAR level for vitamin E is the same for smokers and nonsmokers (1), and therefore, smokers are an appropriate subpopulation for this type of investigation. In addition, there is no upper limit for the age of people for whom the EAR is intended and no restrictions in the cultural and childhood backgrounds (1). Therefore, the ATBC cohort contributes appropriate data for the investigation of the biological relevance of the EAR and the RDA (2000).

The dietary intake of vitamins E and C were estimated at the baseline of the ATBC Study. Evidently, there are changes in the food consumption that occur over the years, whereby some participants increase and some decrease their dietary vitamin E intake. However, it seems unlikely that such changes might be large enough to compromise the validity of this analysis and in any case these changes will reflect what happens in the population as a whole. In the reproducibility study of the food use questionnaire, the findings for vitamin C and E were closely similar (31). Baseline dietary vitamin C intake predicted the effect vitamin E supplementation on mortality over the intervention period, and the effect of vitamin C was not explained by other substances that are present in fruit, vegetables, and berries (20). If there were dramatic changes in vitamin C intake levels, such constancy over years could not occur. There seems no basis to assume that the changes in dietary vitamin E intake could be substantially greater than the changes in vitamin C intake, so that they would make the baseline assessment noninformative for vitamin E.

Finally, the EAR for vitamin E (1) is based on two small laboratory-based studies by Horwitt in the 1950s, in which hydrogen peroxide-induced hemolysis was used as the outcome (2, 3). Thus, the potential limitations of the current study should not be considered in isolation, but should be considered in parallel with the limitations of the Horwitt studies.

### The Effects of Vitamin E on Elderly Deserve Further Research

There is much evidence that oxidative stress increases with age (10–16), and it has been proposed that higher doses of vitamin E might be beneficial for the elderly (17). The reduction in mortality by vitamin E supplementation in the oldest ATBC participants (Table 2, Figure 2) manifested as a longer lifespan. Among 2,284 men with dietary vitamin C intakes above 90 mg/day who smoked less than one pack of cigarettes per day, vitamin E extended lifespan by 2 years at the upper limit of the follow-up age span (73). In addition, vitamin E administration reduced the incidence of pneumonia by 69% in 2,216 males in the ATBC Study who smoked the least and exercised in their leisure time. In those 2,216 male smokers, the cumulative occurrence of pneumonia by the age of 74 years was 19.6% in the no-vitamin E subgroup, but just 6.7% in the vitamin E group. This indicates that 12.9% of participants had no pneumonia by the age of 74 years because of vitamin E administration (69). Evidently, the effects of vitamin E for elderly men should be further examined although negative findings even to the extent of harm have been reported in some contexts.

## Conclusions

This analysis of the ATBC Study found no support for the “estimated average requirement” level of 12 mg/day. The strong evidence of heterogeneity in the effects of vitamin E on mortality in the ATBC Study supports the concept of “individuality in nutrition” so that optimal doses for different population groups can be quite different, depending on age and various life style factors. In several subgroups of the ATBC Study and in several other trials, vitamin E has caused harm at intakes much lower than the stated tolerable upper intake level of 1 g/day. Thus, even though doses in excess of 12 mg/day might be beneficial for some groups of older males, they may also be harmful for some other population groups. Further research is warranted on the effects of vitamins E and C on males over 65 years.

## Data Availability Statement

Datasets generated for this study will not be made publicly available. The dataset was given to the author on the condition that he does not distribute it further. Requests to access the dataset should be directed to Demetrius Albanes albanesd@mail.nih.gov.

## Ethics Statement

This is a secondary analysis of a previously published trial. The participants provided their written informed consent to participate in this study (18, 19).

## Author Contributions

HH planned the study, carried out the analyses, and wrote the manuscript.

### Conflict of Interest

The author declares that the research was conducted in the absence of any commercial or financial relationships that could be construed as a potential conflict of interest.
