# Supplementary material for: Vitamin E and Mortality in Male Smokers of the ATBC Study: Implications for Nutritional Recommendations
Source: Front Nutr. 2020 Mar 31;7:36. doi: 10.3389/fnut.2020.00036 (PMC7136753; doi:10.3389/fnut.2020.00036)
Supplement: Supplementary file 1 [file Data_Sheet_1.PDF]

Vitamin E and mortality in male smokers of the ATBC Study: implications for nutritional recommendations

Harri Hemilä

Department of Public Health
University of Helsinki,
FI-00014 University of Helsinki, Finland.
Email: harri.hemila@helsinki.fi

Supplementary file

submitted to *Frontiers in Nutrition*
https://www.frontiersin.org/journals/nutrition

This supplementary file describes the code by which the statistical calculations have been done.

ver 2020-1-30

Generation of the data set:

```
data deads4;
set oma.hhemila8b;

if treatmgr = "Placebo" or treatmgr = "AT"
   or treatmgr = "BC" or treatmgr = "AT+BC" ;

if vite >0;

if treatmgr = "AT" or treatmgr = "AT+BC" then at_both = 1; else at_both = 0;
if treatmgr = "BC" or treatmgr = "AT+BC" then bc_both = 1; else bc_both = 0;
if treatmgr = "AT+BC" then at_bc = 1; else at_bc = 0;

end = 3237;  ** 12173 - 9132 + 196 (8936);

if deathday <= end and deathday ne . then death = 1;
else death = 0;

if death = 0 then pyrs = (end - randay)/365.25;
if death = 1 then pyrs = (deathday - randay)/365.25;

if age              < 63    then age_n = 50;
if age >= 63 and age < 66    then age_n = 63;
if age >= 66                 then age_n = 66;

if cvitloss >= 90  then cvi_90 = 1; else cvi_90=0;

viteadj   = 0.85*vite;
viteadj80 = 0.80*vite;

if                   viteadj  <9 then vite_n =  8;
if viteadj >=  9 and viteadj <12 then vite_n =  9;
if viteadj >= 12                 then vite_n = 12;

if                     viteadj80  <9 then vite80_n =  8;
if viteadj80 >=  9 and viteadj80 <12 then vite80_n =  9;
if viteadj80 >= 12                   then vite80_n = 12;

run;
```

Calculation of the statistical models:

```
proc freq data=deads4;
table death;
run;

proc means data=deads4;
var vite viteadj;
run;

proc tabulate data=deads4test;
class  at_both;
var       death ;
table   at_both*death death;
run;

*******************  TABLE 1;

proc tabulate data=deads4;
where cvitloss <90;
var      viteadj;
table  viteadj*(median min max N)   ;
run;

proc tabulate data=deads4;
where cvitloss <90;
class  vite_n at_both;
var      viteadj death ;
table  viteadj*median at_both*death N, vite_n    ;
run;

proc sort data=deads4;
by vite_n;
run;
proc phreg data=deads4;
where cvitloss <90;
by vite_n;
model pyrs*death(0)=
at_both
/ties=breslow risklimits;
run;

proc tabulate data=deads4;
where cvitloss <90 and viteadj<12;
class  vite_n at_both;
var       death viteadj;
table  at_both*death N death viteadj*median;
run;

proc phreg data=deads4;
where cvitloss <90 and viteadj <12;
model pyrs*death(0)=
at_both
/ties=breslow risklimits;
run;

proc sort data=deads4;
by vite80_n;
run;
proc phreg data=deads4;
where cvitloss <90;
```

```
by vite80_n;
model pyrs*death(0)=
at_both
/ties=breslow risklimits;
run;

proc phreg data=deads4;
where cvitloss <90 and viteadj80 <12;
model pyrs*death(0)=
at_both
/ties=breslow risklimits;
run;

proc phreg data=deads4;
where cvitloss <90 and age < 63;
by vite_n;
model pyrs*death(0)=
at_both
/ties=breslow risklimits;
run;

proc phreg data=deads4;
where cvitloss <90 and age >= 66;
by vite_n;
model pyrs*death(0)=
at_both
/ties=breslow risklimits;
run;

proc phreg data=deads4;
where cvitloss <90;
model pyrs*death(0)=
at_both
/ties=breslow risklimits;
run;

proc phreg data=deads4;
where cvitloss <90;
model pyrs*death(0)=
vite_AT
at_both
viteadj
/ties=breslow risklimits;
vite_AT =at_both*viteadj;
run;

proc phreg data=deads4;
where cvitloss <90;
model pyrs*death(0)=
at_both
viteadj
/ties=breslow risklimits;
run;


*******************  TABLE 2;


proc tabulate data=deads4;
where cvitloss >=90;
class  vite_n age_n at_both;
var      viteadj death;
```

```
table  viteadj*age_n*median  viteadj*(median min max) age_n*at_both*death
age_n*N, vite_n   ;
run;

proc tabulate data=deads4;
where cvitloss >=90 and viteadj <12;
class  age_n at_both;
var       death;
table   age_n*at_both*death  age_n*N  N ;
run;

proc tabulate data=deads4;
where cvitloss >=90 and viteadj <12 and age < 63;
class  at_both;
var       death viteadj;
table   at_both*death  N viteadj*median;
run;

proc sort data=deads4;
by age_n vite_n;
run;

proc phreg data=deads4;
where cvitloss >=90;
by age_n vite_n;
model pyrs*death(0)=
at_both
/ties=breslow risklimits;
run;

proc phreg data=deads4;
where cvitloss >=90;
by age_n;
model pyrs*death(0)=
at_both
/ties=breslow risklimits;
run;


proc sort data=deads4;
by age_n vite80_n;
run;

proc phreg data=deads4;
where cvitloss >=90;
by age_n vite80_n;
model pyrs*death(0)=
at_both
/ties=breslow risklimits;
run;


proc sort data=deads4;
by age_n;
run;

proc phreg data=deads4;
where cvitloss >=90;
by age_n;
model pyrs*death(0)=
vite_AT
at_both
```

```
viteadj
/ties=breslow risklimits;
vite_AT =at_both*viteadj;
run;

proc phreg data=deads4;
where cvitloss >=90;
by age_n;
model pyrs*death(0)=
at_both
viteadj
/ties=breslow risklimits;
run;


proc phreg data=deads4;
where cvitloss >=90 and viteadj <12 and age < 63;
model pyrs*death(0)=
at_both
/ties=breslow risklimits;
run;


************** vitamin E and BC -interaction    Table 3;

proc tabulate data=deads4;
where  cvitloss >=90 ;
class at_both bc_both vite_n age_n;
var     death ;
table  age_n*at_both*death age_n*N, bc_both   ;
run;

proc sort data=deads4;
by age_n bc_both;
run;

proc phreg data=deads4;
where cvitloss >=90;
by age_n bc_both;
model pyrs*death(0)=
at_both
/ties=breslow risklimits;
run;

proc phreg data=deads4;
where cvitloss >=90;
by age_n;
model pyrs*death(0)=
at_both
bc_both
at_bc
/ties=breslow risklimits;
run;

proc phreg data=deads4;
where cvitloss >=90;
by age_n;
model pyrs*death(0)=
at_both
bc_both
/ties=breslow risklimits;
run;
```

```
*******************        Figure 2;

proc tabulate data=deads4;
class age_n;
var    age ;
table  age_n, (N age*median);
run;

proc tabulate data=deads4;
where cvitloss >=90;
class age_n;
var    age ;
table  age_n, (N age*median);
run;

proc phreg data=deads4;
where cvitloss >=90 ;
model pyrs*death(0)=
age_vite
at_both
age
/ties=breslow risklimits;
age_vite =at_both*(age-50);
run;

proc phreg data=deads4;
where cvitloss >=90 ;
model pyrs*death(0)=
at_both
age
/ties=breslow risklimits;
run;


proc phreg data=deads4;
where cvitloss >=90;
model pyrs*death(0)=
at_both
age63
age66
veage63
veage66
/ties=breslow risklimits;
age63  =(age >= 63 and age < 66);
age66  =(age >= 66);
veage63  =age63*at_both;
veage66  =age66*at_both;
run;

proc phreg data=deads4;
where cvitloss >=90;
model pyrs*death(0)=
at_both
age63
age66
/ties=breslow risklimits;
age63  =(age >= 63 and age < 66);
age66  =(age >= 66);
run;
```
